# Field dataset of the depth to water from diverse wetland types in relation to habitat and soil

**DOI:** 10.1016/j.dib.2024.110656

**Published:** 2024-06-21

**Authors:** Hugo Clément, Guillaume Gayet, Florence Baptist, Jérôme Porteret, Pierre Caessteker, Claire Magand, Anne Vivier, Stéphanie Gaucherand

**Affiliations:** a Laboratoire des écosystèmes et sociétés en montagne (UR 1464), Institut national de recherche pour l'agriculture, l'alimentation et l'environnement, 2 rue de la Papeterie, BP 76, Saint-Martin-d'Hères 38402, France; b Unité d'appui et de Recherche Patrimoine Naturel, Office Français de la Biodiversité, Centre National de la Recherche Scientifique, Muséum national d'Histoire Naturelle, Institut de recherche pour le développement, 36 rue Geoffroy Saint Hilaire, CP 41, Paris 75005, France; c Soltis environnement, 196C rue du Rocher de Lorzier, Moirans 38430, France; d Conservatoire d'espaces naturels de Savoie, Bâtiment Le Prieuré, 165 route de Chambéry, Le Bourget-du-Lac 73370, France; e Direction Acteurs et citoyens, service Usages et gestion de la biodiversité, Office français de la biodiversité, 12 cours Lumière, Vincennes 94300, France; f Direction de la recherche et de l'appui scientifique, Office français de la biodiversité, 12 cours Lumière, Vincennes 94300, France

**Keywords:** Hydrology, Water table, Monitoring wells, Wetland vegetation, Wetland pedology, Groundwater, Data loggers

## Abstract

Wetlands perform important functions and provide essential ecological services, including flood attenuation, groundwater recharge and discharge, and water purification. Human activities such as urban and rural development, drainage, and land alteration can cause major disturbances, often resulting in the drying up of wetlands. Therefore, many restoration projects aim to restore wetland hydrology. Hydrology significantly affects wetland functions by modifying and determining the wetlands physicochemical environment that allows for the development of a specific biota. Despite the importance of hydrology, monitoring efforts are mainly focused on surveying and characterizing wetland habitats or plant composition. There are few datasets available from the monitoring of the depth to water table (DTW) in wetlands and when available they are rarely shared. Collecting hydrological data can contribute to a better understanding of the relationship between hydrology, soil and habitat and can help understand the effect of climate change. From 2021 to 2023, depth to water table, soil and habitat data were collected across a variety of wetland types in France with a focus on hydrological data. Using data loggers placed in 37 monitoring wells across 17 wetlands, 469001 hourly depth to water table and water temperature data were collected. The dataset includes two files containing a total of 22 variables that describe the location of sites, habitat (EUNIS: European Nature Information System), soil hydromorphy, depth to water table, and water temperature. The dataset can be used to better understand wetland hydrology and its relationship to soil and habitat. The data collection process may be used to help restoration project achieve their goal.

Specifications Table

**Table:** 

Subject	Environmental Science: Ecology
Specific subject area	Wetlands hydrology, soil and habitat
Type of data	Table, Raw, Analysed
Data collection	The depth to water and water temperature were collected using monitoring wells equipped with the Rugged TROLL 100 and Rugged BaroTROLL 100 data loggers and transferred to a computer using the In-Situ Win-Situ 5 software. Soil characteristics were established using the Gayet et al. (2023) method [1]. The habitat was determined at a EUNIS 4 level based on vegetation sampling using the quadrat method and the French guide to determining EUNIS terrestrial and marine habitats [2]. Wetland sampled were classified using the hydrogeomorphic (HGM) classification from Brinson [3]. Elevation of monitoring wells were determine using a Trimble® R2 on field or via the Géoportail platform toolbox.
Data source location	• Institution: Institut national de recherche pour l'agriculture, l'alimentation et l'environnement (INRAE) • City/Town/Region: Atlantic, Continental and Alpine French biogeographical regions • Country: mainland France • All data location are provided in the ‘2024-04-02_Dataset_Sites_Habitat_Soil.csv’ dataset.
Data accessibility	Repository name: Recherche Data Gouv Data identification number: https://doi.org/10.57745/DL1NGM Direct URL to data: https://entrepot.recherche.data.gouv.fr/dataset.xhtml?persistentId=doi:10.57745/DL1NGM&faces-redirect=true
Related research article	-

## 1 Value of the Data

• The dataset can be used to describe the hydrological signature of wetlands at a fine scale. It can be used in studies that wish to compare the hydrology of different wetlands around the world.
• The dataset can help engineers and researchers in understanding the relationship between depth to water, soil, and habitat. It can help carry out more in-depth studies of these relationships in different wetlands around the world.
• The dataset is valuable for research on wetland hydrology and its response to global warming.

## 2 Background

In the context of climate change, declining global water resources, and land-use planning leading to the drying up of wetlands, there is an increasing need for knowledge of wetland hydrology. The lack of hydrological data on wetlands is a barrier to a full functional assessment of wetland restoration efforts, as well as to a broader understanding on how wetlands function. Efforts need to be made on the understanding of the linkages between water, soil, vegetation and climate. The collected data will enhance our comprehension of the hydrological processes in wetlands, particularly in relation to their type and location. It will also promote the systematic use of monitoring wells in wetland restoration programs.

## 3 Data Description

The ‘2024-04-02_Dataset_Sites_Habitat_Soil.csv’ dataset (Table 1) provides information on location, elevation, soil characteristics and habitat of the 37 monitoring wells from the 17 wetlands monitored ranging from 51 to 815 m in the continental, Atlantic, and Alpine biogeographical regions of France. The dataset contains 18 variables. The monitored sites included 3 depressional, 20 mineral soil flats, 3 organic soil flat and 11 riverine wetland types.

**Table 1:** Description of the fields in the ‘2024-04-02_Dataset_Sites_Habitat_Soil.csv’ file (DOI: https://doi.org/10.57745/DL1NGM). ntr: nothing to report. For example, if no redox layer was observed when describing the soil, then 'ntr' was used. If a site was not a restored wetland, the restoration year and action were not mentioned and the code 'ntr' was added. na: not available, the data was not collected and is therefore unavailable.

id	identifier for each field observation
site	unique site name
restored	'yes' if it's a restored site; 'no' if it's not a restored site
restoration_year	the year in which the restoration action was initiated
restoration_action	restoration actions that occurred on site
monitoring_well_number	monitoring well number associated with the site name
location	the French department in which the site is located
elevation	ground elevation for each monitoring well (in m)
HGM	hydrogeomorphic type from Brinson (1993) [3], expressed in five categories: ‘riverine or estuarine fringe’, ‘depressional’, ‘slope’, ‘lacustrine fringe’, ‘flat’
EUNIS_habitat_code	code of habitat type according to the EUNIS classification system [4]
EUNIS_habitat_name	name of habitat type (EUNIS level 4) according to the EUNIS classification system [4]
soil_depth	depth of the soil profile (in cm)
soil_redox_depth	depth of occurrence of redox features that covered 5% or more of the soil aggregates (in cm)
soil_redox_thickness	thickness of redox features that covered 5% or more of the soil aggregates (in cm)
soil_reduced_depth	depth of the occurrence of soil horizons with a reduced matrix that covered 95% or more of the soil aggregates (in cm)
soil_reduced_thickness	thickness of soil horizons with a reduced matrix that covered 95% or more of the soil aggregates (in cm)
soil_histic_depth	depth of the occurrence of histic features in the soil profile (in cm)
soil_histic_thickness	thickness of histic features in the soil profile (in cm)

The ‘2024-04-02_Dataset_Dtw_Temp.csv’ dataset (Table 2) contains the hourly depth to water and temperature recordings for each site monitored from February 11, 2021, to March 24, 2023. The dataset contains 4 variables.

**Table 2:** Description of the fields in the ‘2024-04-02_Dataset_Dtw_Temp.csv’ file (DOI: https://doi.org/10.57745/DL1NGM). na: not available.

date	date and time of the data logger depth to water collection
site_monitoring_well	unique site monitoring well name
dtw	depth to water (from ground level to water table level, in cm) data collected by data loggers
temp	water temperature (in Celsius) data collected by data loggers

The ‘2024-04-02_README.txt’ file provides details about the datasets such as general information, file overview, access information and data-specific information.

## 4 Experimental Design, Materials and Methods

In order to sample various types of wetlands occurring in diverse hydrogeological conditions, ninety French organizations involved in wetland restoration were contacted. From the 41 potentially interesting sites suggested, 17 wetlands were chosen based on their geographical location, soil characteristics, habitat, HGM type, and the willingness of local restoration communities to assist in the work. This included sharing maps, historical data, and other information.

**Fig. 1:**
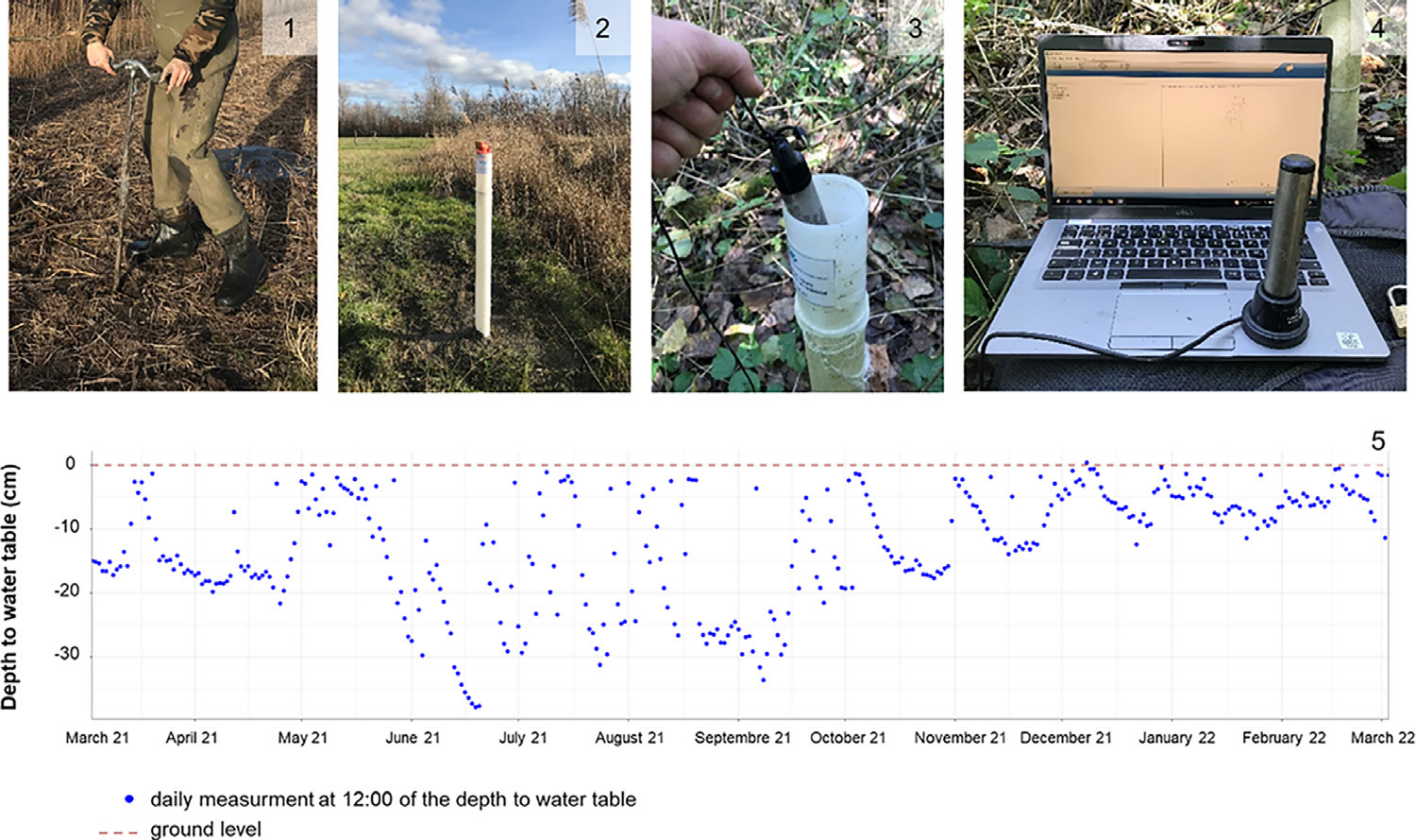
Depth to water table monitoring sequence in a wetland. 1: hand-auger drilling; 2: monitoring well installation; 3: data logger gathering, manual measurement of the depth to water table and maintenance; 4: data USB transfer; 5: daily depth to water table measurement from march 2021 to march 2022. A negative depth to water table means a water table below ground level.

For each site, one to three monitoring wells were installed following the Clément et al. guidance [5]. The number and location of monitoring wells depended on the topographical, soil, and habitat heterogeneity of the site. They have been positioned to best reflect the hydrology of the site. A hand auger was used to dig holes ranging in depth from 1 m to 1.80 m, depending on the type of soil, and obstacles encountered (e.g high amount of stones). The soil and its layers were described visually in the field according to the Gayet et al. [1] methodology, including presence of redox, reduced and histic matrix and the thickness of each layer. Monitoring wells ranged in height from 1 to 2 m. A bottom and top cap were placed on them as well as a filter gauze in order to avoid soil particles entering the monitoring wells through the well screens. Monitoring wells were then placed in the holes previously dug. In each monitoring well, one In-Situ Rugged TROLL data logger (depth to water accuracy: ±0.05% FS; temperature accuracy: ±0.3°C) was hung by an inextensible rope to collect hourly data on the depth to water table and water temperature. For each site, an In-Situ BaroTROLL was installed inside and at the top of a monitoring well to collect barometric pressure needed to get the depth to water table measurement. All monitoring wells were secured with a padlock placed on the top cap. The monitoring well's geographical coordinates at the ground level were recorded using the tool “show coordinates” from the Géoportail toolbox (https://www.geoportail.gouv.fr/carte; horizontal precision: ±1 m) or a GNSS device (Trimble® R2; horizontal precision: ±0.5 m). Projects partners have requested anonymization of the sites and monitoring well coordinates if the data were to be shared. To do so, only the department where the sites are located is indicated in the ‘2024-04-02_Dataset_Sites_Habitat_Soil.csv’ file. Vegetation cover estimates were obtained using quadrats located near each monitoring well. Quadrat size varied in size depending on the habitat sampled as described in Pache [6]. For each monitoring well, the first quadrat was placed in the top right corner of the quadrat next to the monitoring well. The second quadrat was placed randomly in the homogenous vicinity of the monitoring well based on vegetation type. Vegetation sampling was done once the first year of the study at the maximum of vegetation between May and July depending on the site. For each plant species in the quadrat a percentage cover was collected. The habitat was then determined according to the EUNIS classification up to level 4. Data loggers recorded the depth to water table and temperature throughout the year, which were downloaded in the field at least twice a year through USB transfer and the In-Situ Win-Situ 5 software. The hydrological monitoring sequence, from the installation of a monitoring well to the collection of the depth to water table data, is shown in Fig. 1.

## Limitations

The accuracy of monitoring well altitudes may vary from site to site, depending on the calculation methods used. For example, altitudes measured under forest cover are often less accurate than those measured in open areas. Wetlands differ in their conservation state and potential anthropogenic impacts throughout the year. This need to be taking into account when using the datasets.

## Ethics Statement

The authors declare that there are no ethical issues with the data presented and that the ethical requirements for publication in *Data in Brief* has been read and followed

## CRediT authorship contribution statement

**Hugo Clément:** Conceptualization, Methodology, Visualization, Investigation, Validation, Writing – original draft, Writing – review & editing. **Guillaume Gayet:** Conceptualization, Methodology, Validation, Writing – review & editing. **Florence Baptist:** Conceptualization, Methodology, Investigation, Validation, Writing – review & editing. **Jérôme Porteret:** Investigation, Validation, Writing – review & editing. **Pierre Caessteker:** Conceptualization, Validation. **Claire Magand:** Conceptualization, Validation. **Anne Vivier:** Conceptualization, Methodology, Validation. **Stéphanie Gaucherand:** Supervision, Funding acquisition, Conceptualization, Methodology, Investigation, Validation, Writing – review & editing.

## Data Availability

2024-04-02_Dataset_Dtw_Temp (Original data) (Recherche Data Gouv).
